# Prevalence of congenital heart disease in school-aged children and its association with socioeconomic and health service capacity factors: a comparative study of Nepal and China

**DOI:** 10.1186/s12887-026-06897-1

**Published:** 2026-04-21

**Authors:** Kai Bai, Yu Xia, Gajurel Sharad, Teng Wang, Liping He, Yue Gao, Krishna Garu, Yuling Lu, Shuyue Zhang, Lin Duo, Urmila Shakya, Jiang Lu

**Affiliations:** 1 Fuwai Yunnan Hospital, Chinese Academy of Medical Science, Affiliated Cardiovascular Hospital of Kunming Medical University, Kunming, 650102 China; 2 School of Public Health, Kunming Medical University, Kunming, 650500 China; 3 Shahid Gangalal National Heart Center, Kathmandu, Nepal; 4 Annapurna Neuro Hospital, Kathmandu, Nepal

**Keywords:** CHD prevalence, Influencing factors, Socio-economic, Health capacity, Nepal, China

## Abstract

**Background:**

Congenital heart disease (CHD) represents a major contributor to the global burden of childhood disease. However, its burden across diverse socioeconomic and healthcare contexts in Asia remains poorly understood. This study compared CHD prevalence and its associations with socioeconomic conditions and health service capacity among school-age children in Nepal and two Chinese provinces (Yunnan and Xinjiang).

**Method:**

Cross-sectional studies were conducted in Nepal to two provinces in China (Yunnan Province and Xinjiang Uygur Autonomous Region) from June 2024 and May 2025. CHD screening consisted of cardiac auscultation followed by echocardiography for suspected or previously diagnosed cases. Children’s CHD prevalence, socio-economic levels and health service capacity in three regions were compared. Ridge regression was performed at the district/county level to examine the association between socioeconomic and health service capacity factors and the risk of CHD.

**Results:**

A total of 429,204 school-age children were screened, identifying 1,771 children with CHD. Marked interregional differences in CHD prevalence were observed: Nepal exhibited the highest prevalence (5.99 per 1,000 children), followed by Xinjiang (4.51 per 1,000 children) and Yunnan (3.42 per 1,000 children). Substantial disparities in socioeconomic and healthcare indicators were also evident across regions. GDP per capita (USD) ranged from 1,378 in Nepal to 8,751 in Yunnan and 10,071 in Xinjiang, while hospital bed density (per 1,000 population) was 0.39 in Nepal compared to 7.70 and 7.60 in Yunnan and Xinjiang, respectively. Ridge regression analysis revealed that hospital bed density was significantly negatively correlated with CHD prevalence.

**Conclusion:**

Substantial differences in CHD prevalence, socioeconomic conditions, and health service capacity were observed across the three regions. Children’s CHD prevalence was associated with health service capacity, suggesting that greater access to health service capacity may correspond with lower CHD prevalence at the population level.

**Supplementary Information:**

The online version contains supplementary material available at 10.1186/s12887-026-06897-1.

## Introduction

CHD is the most prevalent congenital defect, accounting for nearly one-third of all major congenital anomalies. It is a cardiovascular malformation in young children that is caused by an abnormality in fetal cardiovascular development [1–4]. The prevalence of CHD in the population was estimated to be between 4 and 10 per 1,000 individuals [5–9]. The prevalence of CHD exhibited significant variation on a global scale [10]. The prevalence of CHD was higher in developing countries, particularly in Asia [11]. A 2025 meta-analysis reported a pooled CHD prevalence of 8.9 per 1,000 in Nepal [12], while in India, the prevalence of CHD among children was estimated at 2.25–10.4 per 1,000 [13, 14]. CHD was among the foremost causes of mortality and morbidity among infants and children [15, 16].

A study revealed a higher risk of CHD in children from lower socioeconomic backgrounds [17]. A cohort study in Eastern Europe identified a correlation between low socioeconomic status and cardiovascular disease [18]. Moreover, the World Bank [19] and the United Nations Children’s Fund [20] reported that inequalities in the distribution of external inputs, including education, income, and healthcare, resulted in inequalities in children’s risk of developing CHD [21, 22]. Thus, different socioeconomic risk factors, as well as inequalities in the social determinants of health, may play an important role in the CHD prevalence differences in the regions.

Nepal is classified as a low-income country, which is defined by a low average income and limited access to health resources [23, 24]. In contrast, while Yunnan and Xinjiang are among the less developed provinces in China—characterized by remote geographic location, multi-ethnic composition, and economies that lag behind the more developed central and eastern regions [25, 26], they nonetheless benefit from China’s substantially higher national health expenditure and infrastructure investment, with both socioeconomic and health service capacities exceeding those of Nepal. These marked cross-national differences in development and healthcare infrastructure provide a unique opportunity to examine how contextual factors may relate to CHD burden. In addition, previous studies of CHD prevalence among school-aged children in all three regions [8, 27, 28] reflected CHD prevalence among surviving and diagnosis delays school-aged children. Which were different from prevalence at birth, as it excluded spontaneous closure of minor defects, or mortality in infancy, but often underestimated in low-resource settings due to limited neonatal diagnostic capacity and early mortality [29]. Therefore, conducting community-based screening among school-age children in these regions was essential.

A paucity of epidemiological data exists regarding children diagnosed with CHD in low- and middle-income countries, as compared to high-income countries [30]. Furthermore, the majority of previous studies have focused on a single country, whereas this study comparatively analyzed three regions in two developing countries (China and Nepal). It did so by not only comparing the overall school-based CHD-screened prevalence rate but also analyzing the impact of different socioeconomic and health service levels on the prevalence of CHD. This analysis will help to better understand the impact of socioeconomic and health service capacity on CHD and provide basic data for in-depth related risk factor research of CHD prevention. The study will guide the optimization of medical resource equity.

## Methods

### Study design and participants

Since 2017, the Yunnan Provincial Government, Fuwai Yunnan Hospital, Chinese Academy of Medical Sciences (Fuwai Yunnan Hospital) have carried out CHD screening for school-age children under the Yunnan Provincial Comprehensive Prevention and Control of Congenital Heart Disease among Children Plan. In addition, Fuwai Yunnan Hospital, as the National Regional Cardiovascular Center, was also authorized and responsible for the service radiating to neighboring provinces and countries. The joint CHD screening activity in Nepal was based on the Memorandum of Understanding between Fuwai Yunnan Hospital, China, and Shahid Gangalal National Heart Center, Nepal.

In this multi-center cross-sectional study conducted from June 2024 to May 2025, a total of 10 counties/districts were included. In China, Yunnan Province and Xinjiang Uyghur Autonomous Region were purposively selected based on their geographic remoteness, multi-ethnic composition, and below-average socioeconomic and health service capacity. Within Yunnan, five counties (Jiangcheng, Jinggu, Zhenyuan, Jingdong, and Mojiang) were included; within Xinjiang, three counties (Akesu, Wensu, and Awati) were included. All children aged 3–18 years enrolled in schools in these areas were invited to participate. In Nepal, two districts (Dolakha and Sindhupalchowk) were selected based on feasibility and resource constraints. Within these districts, a convenience sample of schools was recruited, and all enrolled children in the participating schools were included in the study. A total of 429,204 children and adolescents completed the screening and were included in the final analysis. Exclusion criteria: Individuals were excluded from the study if they: (1) were diagnosed with or found to have acquired heart diseases (e.g., rheumatic heart disease, cardiomyopathy, infective endocarditis) during screening echocardiography; (2) did not provide written informed consent from a parent or guardian.

## Data collection

### Basic information collection

Information on children by sex and age was collected by school teachers or county/district workers at the screening sites. Socioeconomic and health service capacity data for Yunnan Province and Xinjiang Uygur Autonomous Region were obtained from the China Statistical Yearbook 2024 and the National Economic and Social Development Statistical Bulletin 2024 [31], and for Nepal from the World Development Bank Report [32]. These data were used to describe the contextual characteristics and regional disparities across the three study settings. Socioeconomic indicators include: per capita GDP (current US$), per capita disposable income (current US$), per capita healthcare expenditure (current US$), and rural population (%). Health service capacity includes: the number of hospital beds, doctors, nurses, and midwives/1000 people, maternal mortality rate (national estimate, per 100,000 live births), mortality rate, infant (per 1,000 live births), and life expectancy at birth(years). All indicators were presented at the provincial (China) or national (Nepal) level for descriptive comparison only.

In addition, For the five counties in Yunnan Province and the three counties in Xinjiang Uygur Autonomous Region, all variables used in the correlation and ridge regression analyses-including GDP per capita, per capita disposable income, hospital beds per 1,000 population, physicians per 1,000 population, rural population proportion, and altitude-were collected at the county level. For Nepal, only national-level estimates were available for these indicators; thus, both districts (Dolakha and Sindhupalchowk) were assigned the same national values. We acknowledge that the assignment of national-level data to Nepalese districts may obscure within-country heterogeneity and attenuate true associations; findings should therefore be interpreted with appropriate caution.

### Clinical information collection

The same screening team used the same type of echo with the same protocol. The screening team was divided into two groups, each staffed with at least one team leader, one cardiologist, one cardiac ultrasound physician, eight to ten paramedic staff and a volunteer. Both groups operated concurrently to conduct the screenings (2 more cardiologists from the local heart center joined the team in Nepal). First, each subject was physically examined by an experienced cardiologist, with special attention to growth and cyanosis, followed by cardiac auscultation, and only for subjects with significant growth retardation, cyanosis, or murmurs(Murmurs were defined as those graded ≥ 2/6 in intensity or with holosystolic, diastolic, or continuous characteristics), a cardiac sonographer used a portable ultrasound machine (Philips X50 ^®^) for examination. Volunteers recorded diagnoses according to ICD-10-CM (International Classification of Diseases, 10th Revision); specific ICD-10-CM information is provided in the supplementary material. Complex CHD included the following categories: severe pulmonary stenosis with significant sub-valvular obstruction and right-to-left interatrial shunting, tetralogy of Fallot, transposition of the great arteries with atrioventricular disharmony and ventriculo-arterial disharmony, and hypoplastic left heart syndrome [33]. Two or more CHD was defined as the coexistence of two or more simple congenital heart defects in the same patient, for example, atrial septal defect (ASD) with ventricular septal defect (VSD). Complex congenital malformations involving a single primary diagnosis (e.g., tetralogy of Fallot) were not classified under this category.

Children diagnosed with CHD were categorized by cardiac ultrasonographers and cardiologists based on the results of the examination. The categories were defined as follows: Not needing treatment included specific anatomical characteristics such as a Patent Foramen Ovale with a resting diameter < 2 mm or an Atrial Septal Defect with a diameter < 5 mm and insufficient rim, absence of related symptoms or history of paradoxical embolism, and normal cardiopulmonary function without evidence of right ventricular volume overload; Needing treatment comprised presence of related symptoms, objective evidence of right ventricular volume overload, or a clinically significant defect size; postoperative referred to patients who had undergone surgical or interventional treatment and required subsequent follow-up; Regular follow-up was needed included patients with a indication for intervention or surgeon treatment, but in whom immediate treatment was contraindicated, typically due to low age and weight, severe pulmonary hypertension, warranting optimized medical therapy and close surveillance until clinically suitable for the procedure.

This study was conducted in accordance with the requirements of the Declaration of Helsinki, and informed consent was obtained from the legal guardians or parents of each enrolled child.

### Statistical analysis

Categorical variables were described by frequency and percentage (%). The prevalence of CHD and subtypes was expressed per 1,000 people. Differences in the prevalence of CHD in different regions were analyzed using the chi-square test or Fisher’s exact test. Ridge regression was employed to examine the association between socioeconomic and health service capacity factors and CHD risk, given substantial multicollinearity among these ecological-level predictors (see Supplementary Materials for collinearity diagnostics). This method was considered more appropriate than alternatives (e.g., LASSO, principal component regression) for the present study, as it retains all theoretically relevant variables while stabilizing coefficient estimates through L2 regularization [34]. Analyses were conducted using R software (version 4.4.3). A two-sided p-value of less than 0.05 was considered statistically significant.

## Results

### CHD screening and prevalence in 3 different regions

A total of 429,204 children aged 3–18 years were screened across 43 schools in Nepal, 355 schools in Yunnan, and 1,163 schools in Xinjiang, identifying 1,771 children with CHD. In Nepal, 8,854 children from Dolakha and Sindupalchowk districts were screened, yielding 53 CHD cases (prevalence: 5.99 per 1,000 children). In Yunnan, 162,668 children were screened across five counties (Jiangcheng, Jinggu, Zhenyuan, Jingdong, and Mojiang), identifying 556 CHD cases (3.42 per 1,000 children). In Xinjiang, 257,682 children were screened across three counties (Aksu, Awati, and Wensu), identifying 1,162 CHD cases (4.51 per 1,000 children). CHD prevalence varied significantly across the three regions, with the highest rate observed in Nepal and the lowest in Yunnan (*P* < 0.01). In all regions, CHD prevalence was higher among girls than boys. Significant differences in CHD prevalence by sex and age group were also observed between Yunnan and Xinjiang (*P* < 0.05, Table 1).

**Table 1:** Prevalence and gender and age distribution of congenital heart disease (CHD) in 3 different regions

Age, yr	Nepal (*n* = 8854)			Yunnan (China) (*n* = 162668)			Xinjiang (China) (*n* = 257682)			*P* value
	*n*	CHD	per 1,000 children (95‰ CI)	*n*	CHD	per 1,000 children (95‰CI)	*n*	CHD	per 1,000 children (95‰ CI)	
Boys										
< 6	427	4	9.37 (2.56,23.81)	8606	27	3.14 (2.07,4.56)	10,160	42	4.13 (2.98,5.58)	< 0.01
6–12	2729	15	5.50 (3.08,9.05)	44,431	156	3.51^#^ (2.98,4.11)	74,700	360	4.82^#^ (4.34,5.34)	< 0.01
> 12	1587	5	3.15 (1.02,7.34)	29,096	69	2.37 (1.85,3.00)	45,152	155	3.43 (2.91,4.02)	< 0.05
*P* value			0.24			< 0.05			< 0.01	
Girls										
< 6	507	0	0 (0,7.25)	8090	35	4.33 (3.02,6.01)	9161	48	5.24 (3.87,6.94)	0.23
6–12	2420	20	8.26 (5.06,12.74)	41,955	182	4.34 (3.73,5.01)	73,872	392	5.31 (4.80,5.86)	< 0.01
> 12	1184	9	7.60 (3.48,14.38)	30,490	87	2.85^*^ (2.29,3.52)	44,637	165	3.70^*^ (3.15,4.30)	< 0.01
*P* value			0.09			< 0.01			< 0.01	
Both										
< 6	934	4	4.28 (1.17,10.93)	16,696	62	3.71 (2.85,4.76)	19,321	90	4.66 (3.75,5.72)	0.37
6–12	5149	35	6.80 (4.74,9.44)	86,386	338	3.91 (3.51,4.35)	148,572	752	5.06 (4.71,5.44)	< 0.01
> 12	2771	14	5.05 (2.76,8.46)	59,586	156	2.62^*^ (2.22,3.06)	89,789	320	3.56^*^ (3.18,3.98)	< 0.01
*P* value			0.49			< 0.01			< 0.01	
Total	8854	53	5.99* (4.49,7.82)	162,668	556	3.42 (3.14,3.71)	257,682	1162	4.51 (4.25,4.78)	< 0.01

^*^compared with other groups, *P* < 0.05; ^#^Compared with > 12 groups, *P* < 0.05

### Characteristics of CHD

Among the 1,771 CHD cases identified across the three regions, the mean age of affected children was 10.38 ± 0.45 years in Nepal, 10.07 ± 0.16 years in Yunnan, and 10.48 ± 0.10 years in Xinjiang. The distribution of CHD subtypes varied by region (*P* < 0.01). In Nepal, atrial septal defect (ASD) was the predominant subtype, accounting for 18 cases (34.62%). In contrast, patent foramen ovale (PFO) was the most frequent finding in Yunnan (122 cases, 37.08%) and Xinjiang (173 cases, 33.40%). Xinjiang had the highest proportion of children with two or more CHD types (128 cases, 24.71%), while Nepal exhibited the highest prevalence of complex CHD (3.84%). Regarding treatment recommendations and follow-up, Nepal had the highest proportion of children recommended for further intervention (27 cases, 50.94%). However, the proportion of children who had already received post-CHD treatment was markedly higher in Yunnan (227 cases, 40.83%) and Xinjiang (644 cases, 55.42%) compared to Nepal (1.89%) (*P* < 0.01, Table 2).

**Table 2:** Ultrasound findings and pattern of congenital heart disease (CHD) and age in 3 different regions

	Nepal *n*(%)	Yunnan *n*(%)	Xinjiang *n*(%)	*P* value
Gender				< 0.01
Boys	24(45.28)	252(45.32)	557(47.93)	
Girls	29(54.72)	304(54.68)	605(52.07)	
Age				0.20
< 6	4(7.55)	62(11.15)	90(7.74)	
6–12	35(66.03)	338(60.79)	752(64.72)	
> 12	14(26.42)	156(28.06)	320(27.54)	
Ultrasound findings				< 0.01
Regular follow-up was needed	1(1.89)	8(1.44)	16(1.38)	
Needing treatment	27(50.94)	70(12.59)	168(14.46)	
Postoperative (previously treated)	1(1.89)	227(40.83)	644(55.42)	
No needing treatment	24(45.28)	251(45.14)	334(28.74)	
Disease types in children with non-operative CHD				< 0.01
Patent foramen ovale (PFO)	11(21.15)	122(37.08)	173(33.40)	
Atrial septal defect (ASD)	18(34.62)	43(13.07)	66(12.74)	
Ventricular septal defect (VSD)	6(11.54)	15(4.56)	44(8.49)	
Aortic regurgitation (AR)‌	2(3.85)	28(8.51)	24(4.64)	
Patent ductus arteriosus (PDA)	3(5.77)	15(4.56)	17(3.28)	
Pulmonary stenosis (PS)	1(1.92)	13(3.95)	9(1.74)	
Mitral valve prolapse (MVP)	1(1.92)	8(2.44)	9(1.74)	
Others	3(5.77)	36(10.94)	46(8.88)	
Two or more CHD	5(9.62)	43(13.07)	128(24.71)	< 0.01
Complex CHD	2(3.84)	6(1.82)	2(0.38)	< 0.05

Note: Some children presented with more than one congenital heart defect. Percentages for CHD subtypes were calculated based on the total number of patients. “Two or more CHD” was defined as the coexistence of two or more simple cardiac defects in the same patient (e.g., atrial septal defect with ventricular septal defect). Complex congenital malformations involving a single primary diagnosis (e.g., tetralogy of Fallot) were not included in this category

### Comparison of health service capacity and socio-economic status in different regions

**Fig. 1:**
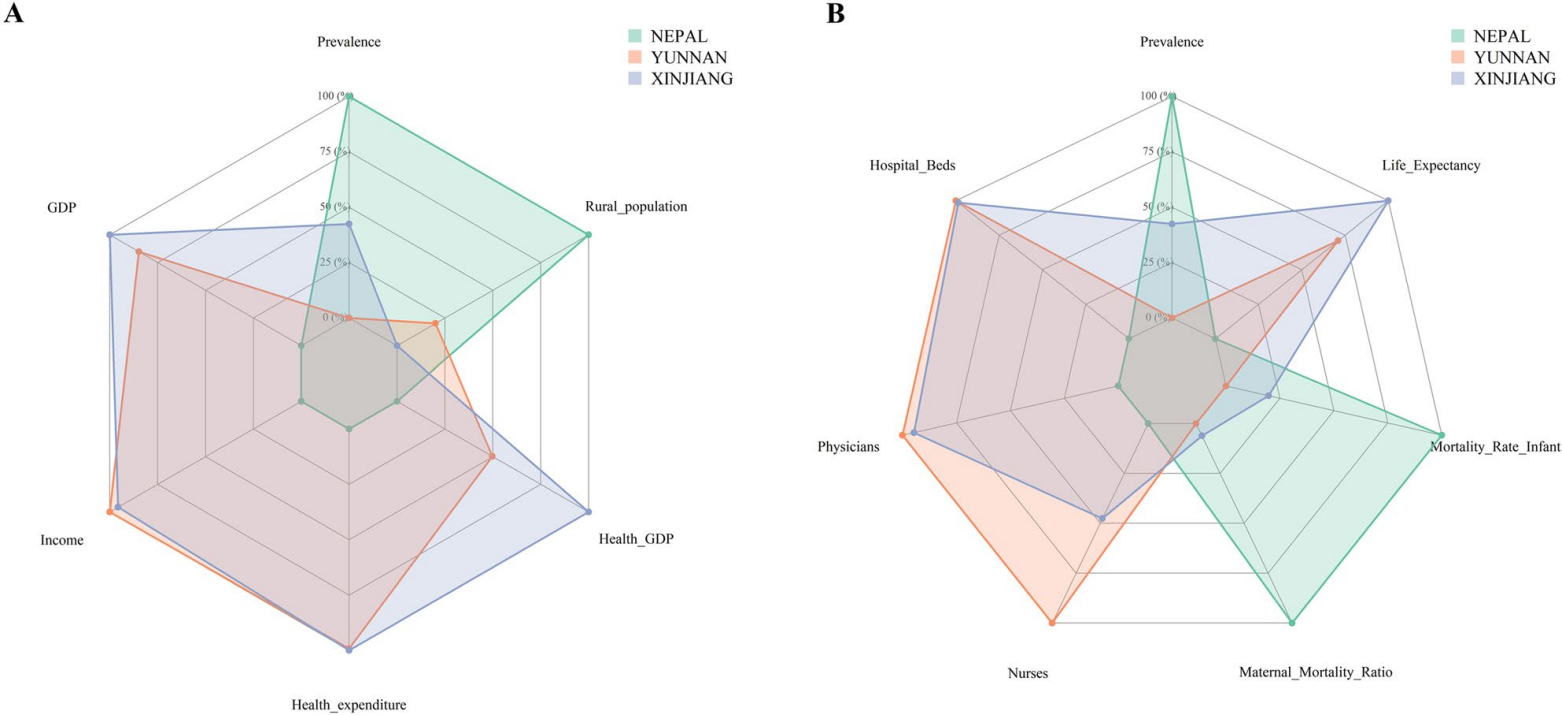
Comparison of socioeconomic and health service capacity with CHD prevalence in three regions. **A** Comparison of different socioeconomic indicators and CHD prevalence in three regions. **B** Comparison of health service capacity and CHD prevalence in three regions

Marked disparities in health service capacity and socioeconomic conditions were observed across the three regions. Hospital bed density (per 1,000 population) was lower in Nepal (0.39) compared to Yunnan (7.70) and Xinjiang (7.60). Similarly, physician density (per 1,000 population) in Nepal (0.87) was considerably lower than in Yunnan (3.14) and Xinjiang (3.02). In contrast, nurse and midwife density was relatively comparable across regions. Socioeconomic indicators revealed similar patterns: GDP per capita (USD) in Nepal (1,377.63) was substantially lower than in Yunnan (8,751.37) and Xinjiang (10,071.04). Despite Nepal allocating 5.42% of its GDP to health expenditure—approaching the levels in Yunnan (7.76%) and Xinjiang (10.5%)-its rural population proportion (78%) was markedly higher than in Yunnan (50%) and Xinjiang (43%) (Table 3; Fig. 1). County-level comparisons yielded consistent findings, with Nepal demonstrating the most vulnerable health service capacity and socioeconomic status across all indicators (Table S1).

**Table 3:** Comparison of health service capacity and socio-economic factors in different regions

Health service capacity and socio-economic factors	Nepal	China	Yunnan	Xinjiang
Health service capacity				
Hospital beds (per 1,000 people)	0.39	7.23	7.7	7.60
Physicians (per 1,000 people)	0.87	3.40	3.14	3.02
Nurses and midwives (per 1,000 people)	3.50	4.00	4.36	3.91
Maternal mortality ratio (national estimate, per 100,000 live births)	142.00	15.10	9.70	17.89
Mortality rate, infant (per 1,000 live births)	23.00	4.80	2.75	6.75
Life expectancy at birth, total (years)	70.48	78.587	74.40	76.00
Socio-economic capacity				
GDP per capita (current US$)	1377.63	12614.06	8751.37	10071.04
Per capita disposable income (current US$)	1005.68	5639.86	4086.08	3951.61
Current health expenditure per capita (current US$)	65.00	670.51	404.89	407.62
Current health expenditures (% of GDP)	5.42	7.13	7.76	10.12
Rural population (% of total population)	78	34	50	43

Note: Current health expenditure per capita: Total health expenditure (public and private) divided by total population, in current U.S. dollars. Current health expenditure (% of GDP): Level of current health expenditure expressed as a percentage of GDP. Rural population (% of total population): People living in rural areas as defined by national statistical offices

### Correlation analysis

**Fig. 2:**
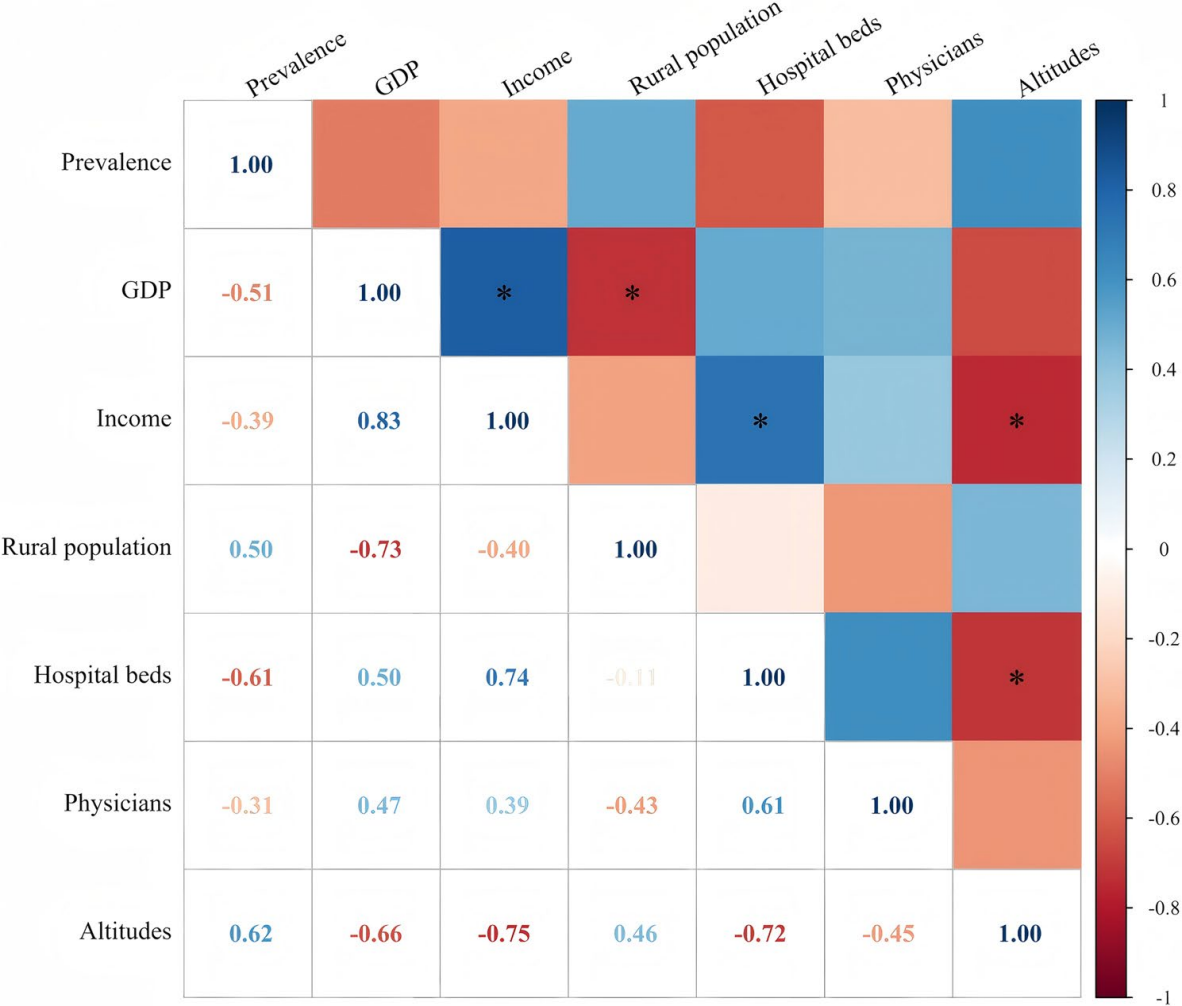
Correlation analysis of socio-economic, health service capacity, and CHD prevalence

County-level correlation analysis revealed that GDP per capita, per capita disposable income, hospital bed density, and physician density were negatively associated with CHD prevalence, while rural population proportion and altitude were positively associated. However, none of these correlations reached statistical significance (*P* > 0.05, Fig. 2), suggesting that multicollinearity among variables may have obscured underlying associations. To address this, ridge regression was employed to further examine the relationships between these contextual factors and CHD prevalence at the district/county level.

### The potential impact of healthcare capacity and socioeconomic factors on congenital heart disease in children

**Fig. 3:**
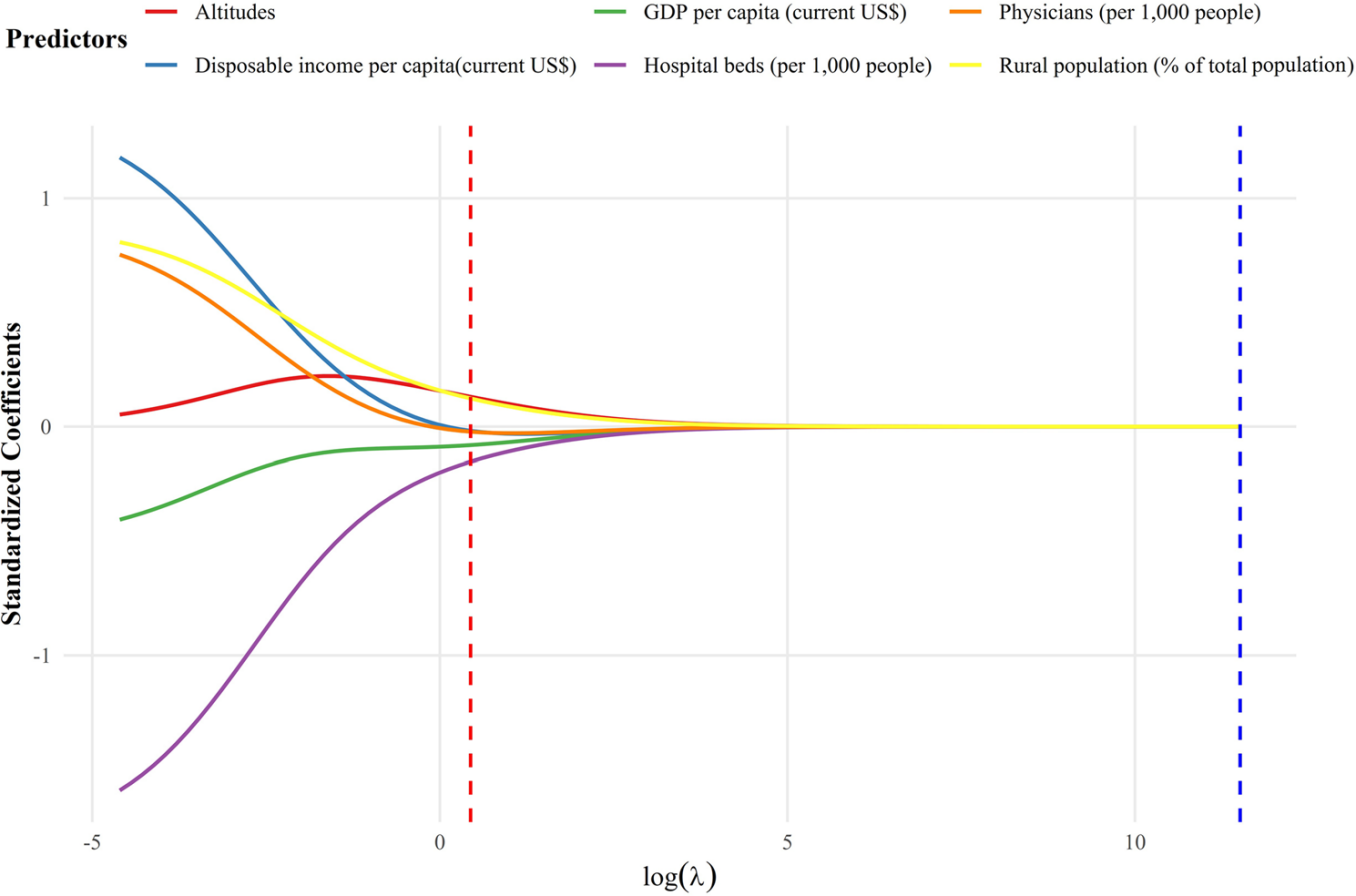
The relationship between the standardized regression coefficients of different variables and log (λ) in ridge regression

**Fig. 4:**
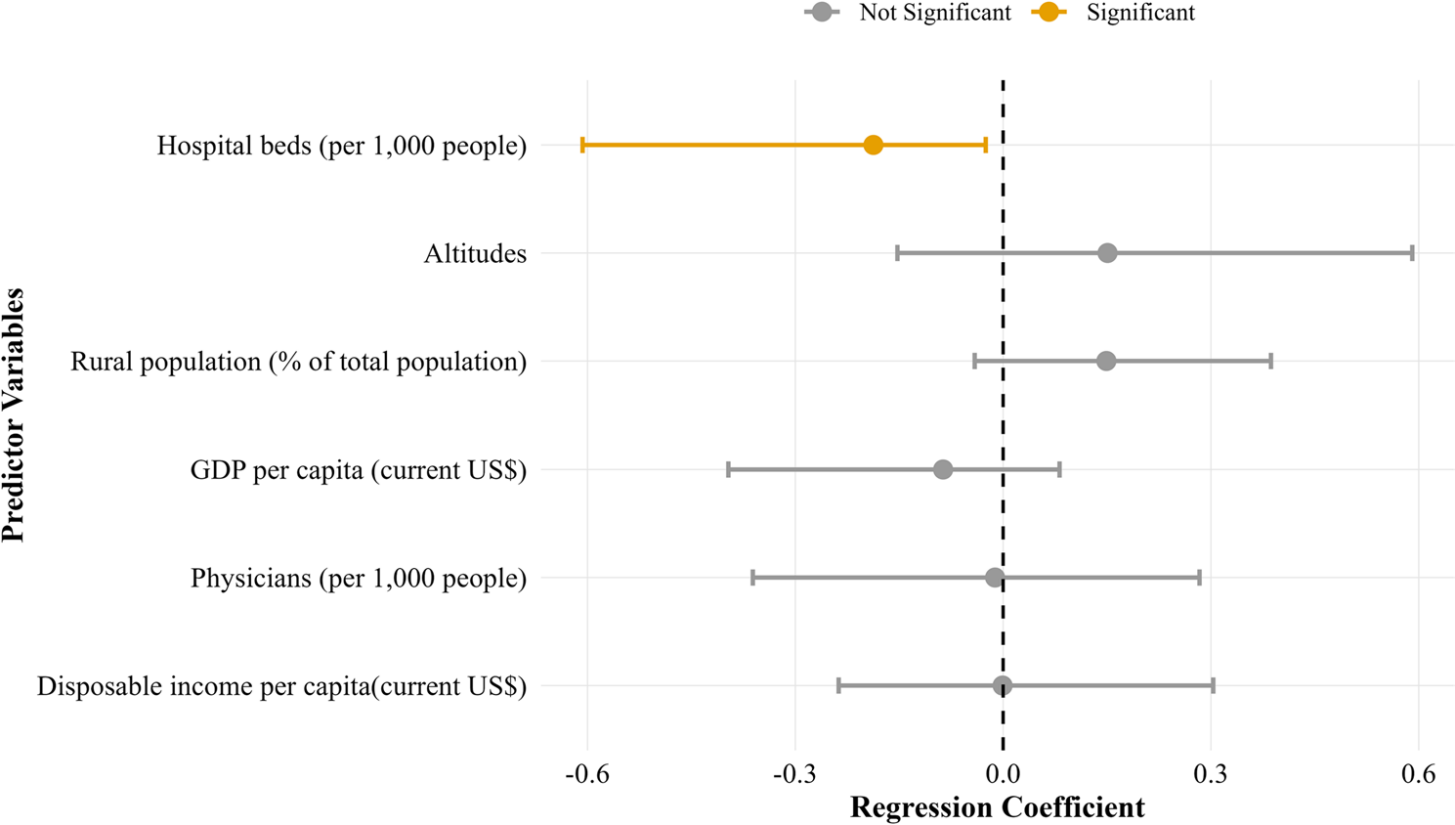
Ridge regression coefficient estimates with 95% confidence intervals

The optimal lambda (λ) value for ridge regression was estimated using maximum likelihood estimation under k-fold cross-validation, minimizing the mean square error (MSE). The optimal λ was 1.835 (Figure S2). The ridge plot (Fig. 3) illustrates the relationships between predictors and CHD prevalence at this optimal λ value. Rural population proportion and altitude were positively associated with CHD prevalence, whereas GDP per capita, per capita disposable income, hospital bed density, and physician density were negatively associated. Among these, hospital bed density (per 1,000 population) exhibited the strongest negative association with CHD prevalence in school-age children. Coefficient estimates with 95% confidence intervals were subsequently calculated. Hospital beds per 1,000 population (β = -0.275, 95% CI: -0.512 to -0.038) demonstrated a statistically significant association with CHD prevalence (Table 4; Fig. 4).

**Table 4:** Ridge regression coefficients and standardized regression coefficients for the effect of socioeconomic and health service capacity on the prevalence of CHD

Factor	Regression coefficient	Standardized regression coefficient
Hospital beds (per 1,000 people)	-0.27454708	-0.1482842
Altitude	0.22099691	-0.1277574
Rural population (% of total population)	0.21853043	-0. 1,198,771
GDP per capita (current US$)	-0.12698036	-0.08001346
Physicians (per 1,000 people)	-0.01700760	-0.02344682
Per capita disposable income (current US$)	-0.00110518	-0.02041086

## Discussion

This large-scale cross-sectional study of 429,204 school-aged children in Nepal and two Chinese provinces (Yunnan and Xinjiang) revealed three principal findings. First, marked geographic disparities in CHD prevalence were observed, with Nepal exhibiting the highest prevalence (5.99 per 1,000 children), followed by Xinjiang (4.51 per 1,000) and Yunnan (3.42 per 1,000). Second, CHD prevalence was consistently higher among girls than boys across all three regions, and significant differences in defect type distribution were evident—ASD predominated in Nepal, while PFO was most frequent in Yunnan and Xinjiang. Third, ridge regression analysis identified hospital bed density as the strongest negative correlate of CHD prevalence at the district/county level, suggesting that health service capacity may play a critical role in shaping population-level CHD burden.

Nepal’s CHD prevalence shows a significant increase from 1.3per 1,000 children previously reported in Kathmandu (Bahadur et al.) [28] and ASD dominance (34.62%) was similar to A meta-analysis done in Nepal [24]. The prevalence discrepancy may be related to improvements in on-site ultrasound machine diagnosis and the screening by joint Nepal-Chinese screening teams. The prevalence of CHD in Yunnan was comparable to the findings of Genghao Q [35] and in Xinjiang, CHD prevalence was lower than the previously reported 16.5 per 1,000 children prevalence [8]. This decline may reflect several interrelated factors: substantial improvements in socioeconomic status and population health over the past decade [27], which likely reduced maternal exposure to CHD risk factors during pregnancy; enhanced health service capacity and prenatal screening capacity, potentially leading to termination of severe cases detected in utero; and changes in diagnostic criteria or case ascertainment methods over time, which may limit direct comparability with historical data. These factors underscore the need for standardized protocols in future studies. The higher prevalence of CHD identified in girls than in boys in the three regions was consistent with a retrospective study in Canada that reported a significantly higher prevalence of CHD in females among children and adults [36] and warranted further investigation.

Several mechanisms may explain the observed regional disparities in CHD prevalence, particularly the higher burden in Nepal compared to Yunnan and Xinjiang. Health system access emerges as a critical factor: Despite the GDP per capita in Nepal (1,377 USD) being much lower than in Yunnan/Xinjiang, Nepal allocated 5.42% of its GDP to health expenditure (comparable to Yunnan/Xinjiang’s 7.76%–10.5%), but the high rural population (78%) and mountainous terrain in Nepal likely impeded service delivery. Nepal’s physician (0.87/1,000) and bed ratios (0.39/1,000) were about < 15% of Yunnan/Xinjiang regions’ capacity. A comparison of Yunnan and Xinjiang reveals that the difference between the two was negligible. Yet, CHD post-treatment rates in the remote community schools were critically low (1.89%) in Nepal, indicating severe gaps related to socio-economic and health service capacity, compared with higher CHD post-treatment rates in Yunnan/Xinjiang (40.83%–55.42%). Economically disadvantaged regions, characterized by higher altitudes, often face limitations in healthcare resources and infrastructure. May reflect multi-factorial influences, including limited prenatal care, higher exposure to environmental teratogens, and genetic factors [37–39], aligned with global patterns where lower-resource settings exhibit elevated CHD rates [17, 40]. This finding suggests a potential indirect effect of district health service capacity on the prevalence of CHD, possibly through its influence on antenatal services.

Nepal’s unique socioeconomic and geographic context also provided essential background for interpreting these disparities. Previous studies have documented higher CHD prevalence at high altitudes, potentially due to chronic maternal hypoxia during pregnancy affecting fetal cardiovascular development [41, 42]. While the average altitude in our study Nepal districts (approximately 1,500-3,000 m) was broadly comparable to parts of Yunnan and Xinjiang, the interaction of altitude with limited antenatal care access in Nepal may amplify its effects. As a low-income country with a per capita GDP of approximately US$1,378—roughly one-seventh that of Yunnan and one-eighth that of Xinjiang—Nepal faced fundamental constraints on health system investment. The Nepal mountainous topography, particularly in the sampled districts of Dolakha and Sindhupalchowk, created physical barriers to healthcare access that compound economic limitations. These districts were also severely affected by the 2015 earthquake, which damaged health infrastructure and disrupted service delivery, with lingering effects on health system capacity. Furthermore, the high proportion of rural population (78%) means that most residents depended on peripheral health posts with limited diagnostic capabilities, rather than the centralized hospital systems available in urban areas. This geographic and economic context explains why, despite comparable health expenditure as a percentage of GDP, Nepal’s health service capacity indicators lagged so substantially behind the Chinese provinces, and the potential reason CHD detection and treatment rates remain low.

Our study identified multicollinearity in the multiple linear regression model, as evidenced by the tolerance and variance inflation factors. This multicollinearity resulted in instability in the calculated coefficients and confusion in the direction. To address this, ridge regression was employed as it provides more stable estimates by introducing a small amount of bias into the regression coefficients [43]. This approach revealed that hospital bed density was the strongest negative correlate of CHD prevalence among the variables examined. This finding aligns with previous studies reporting higher CHD birth prevalence in areas with fewer healthcare facility beds [44], potentially reflecting the role of healthcare infrastructure in facilitating access to prenatal screening and detection of life-threatening anomalies via fetal ultrasound [45].

These findings carried significant implications for health equity and resource allocation. The stark disparities between Nepal and the Chinese regions—particularly the 30-fold difference in post-treatment rates—underscored the need to strengthen health systems in underserved settings. Our finding by ridge regression also reflected that hospital bed density was the strongest negative correlate of CHD prevalence suggested that strategic infrastructure investments may yield substantial public health benefits. For Nepal and similar low-resource settings. From a health equity perspective, our study indicated that reducing CHD disparities required moving beyond a narrow focus on clinical services to address the broader social determinants of health. The World Bank and UNICEF have documented how inequalities in education, income, and healthcare access translate into inequalities in children’s health outcomes [19, 20]; our findings extended this evidence base specifically to CHD. For China, while Yunnan and Xinjiang lagged behind the country’s more developed eastern provinces, the health system capacity substantially exceeds Nepal’s, demonstrating that economic development coupled with strategic health investments could reduce CHD burden even in geographically remote, multi-ethnic regions. This offered lessons for other low- and middle-income countries seeking to strengthen their cardiac care infrastructure.

The study has some limitations. First, due to the cross-sectional ecological design, this study is limited to describing correlations between population-level CHD prevalence and area-level characteristics, such as socioeconomic conditions and health service capacity. It cannot infer causal relationships, underscoring the need for future longitudinal cohort studies. Secondly, the Nepal sample was limited to two districts, the findings only reflect the CHD burden in these specific settings and should not be generalized to Nepal as a whole. Moreover, regarding cross-country comparisons between Nepal and China. These include: differences in screening quality, diagnostic capacity and referral pathways between the two settings, Nepal’s substantially smaller sample size relative to China, which may affect statistical power and the stability of coefficient estimates; and heterogeneity in the types of schools included and underlying population structures. These contextual factors may confound direct comparisons and warrant cautious interpretation. Finally, since specific socioeconomic and health service capacity for each township in Nepal could not be found, only six independent variables were included in the ridge regression, rather than the 11 listed in Table 3. Moreover, it is important to note that ridge regression yields biased coefficient estimates; therefore, the magnitude of these estimates should be interpreted as indicators of relative association rather than causal effects.

Despite these limitations, this study represents one of the largest cross-national comparisons of school-based CHD prevalence in Asia, encompassing over 429,000 children across diverse geographic and socioeconomic settings. The standardized screening protocol, using the same equipment, diagnostic criteria, and training across all sites, strengthens confidence in the comparability of findings. Future research should prioritize longitudinal cohort studies. Additionally, implementation research evaluating the effectiveness and cost-effectiveness of different screening delivery models, school-based versus integrated into maternal and child health programs, could guide scale-up strategies in resource-constrained settings.

## Conclusion

This study reveals significant disparities in CHD burden across Nepal, Yunnan, and Xinjiang, with children in Nepal disproportionately affected-a finding that aligns with the region’s markedly lower health service capacity (physician and hospital bed density) and socioeconomic conditions compared to the two Chinese provinces. Hospital bed density emerged as the strongest negative correlate of CHD prevalence, suggesting that health service capacity may play a critical role in shaping disease burden. These findings carry implications for policy and practice: strengthening health systems in underserved regions through expanding hospital capacity and screening programs could improve early detection and reduce CHD disparities. The pronounced cross-regional differences underscore the need for equity-oriented health policies that prioritize vulnerable populations in low-resource settings. Due to the cross-sectional ecological design, causality cannot be inferred. Future longitudinal studies with individual-level data and district-level analyses are warranted to validate these findings and inform targeted interventions.

## Supplementary Information

Supplementary Material 1.

## Data Availability

The data that support the findings of this study are available from the corresponding author upon reasonable request.
