# Supplementary material for: Prevalence of congenital heart disease in school-aged children and its association with socioeconomic and health service capacity factors: a comparative study of Nepal and China
Source: BMC Pediatr. 2026 Apr 21;26:520. doi: 10.1186/s12887-026-06897-1 (PMC13235184; doi:10.1186/s12887-026-06897-1)
Supplement: Supplementary file 1 — Supplementary Material 1. [file 12887_2026_6897_MOESM1_ESM.docx]

Supplementary Material

Supplementary Figures


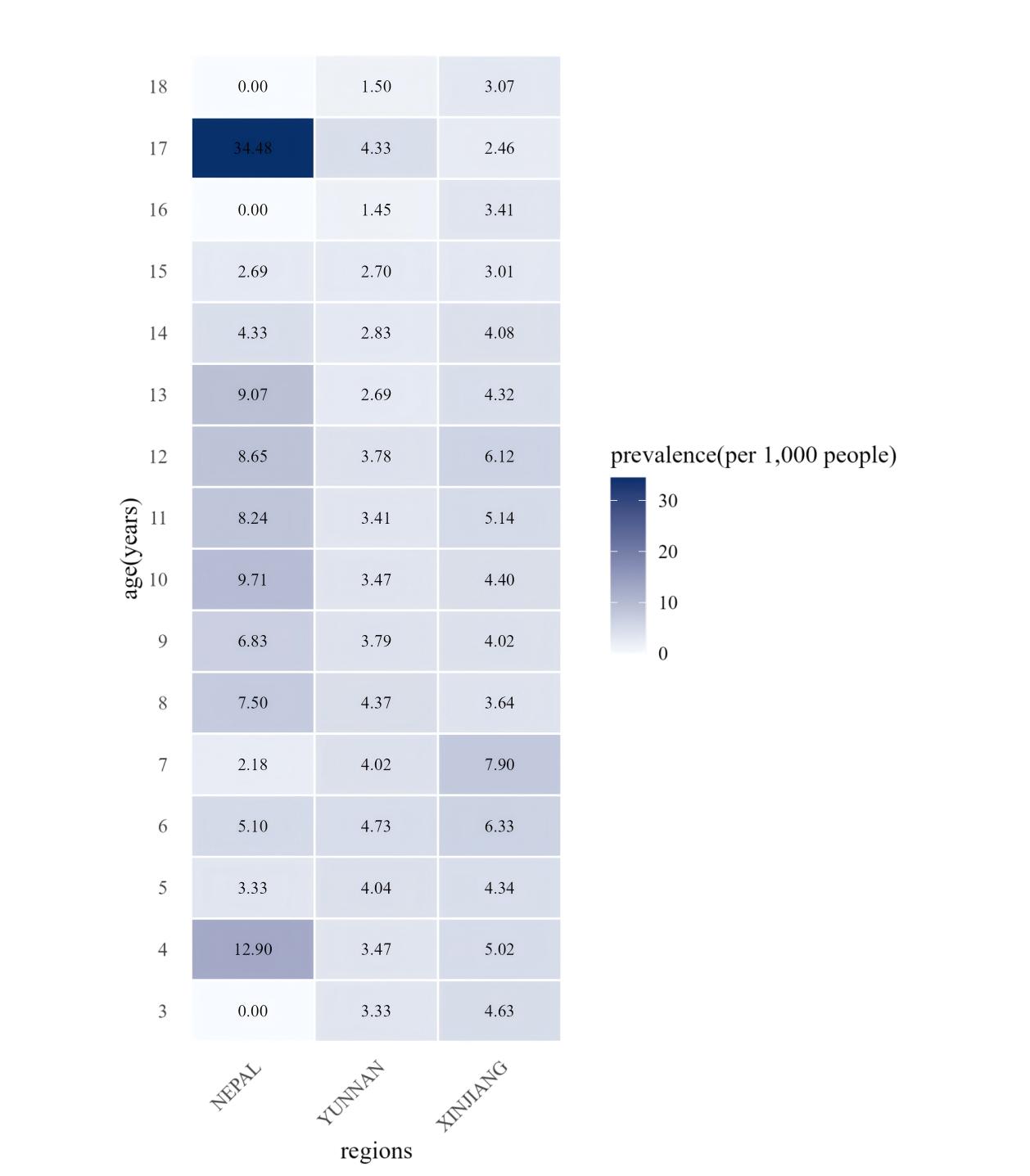


**Figure S1** Distribution of CHD prevalence by age in three regions.


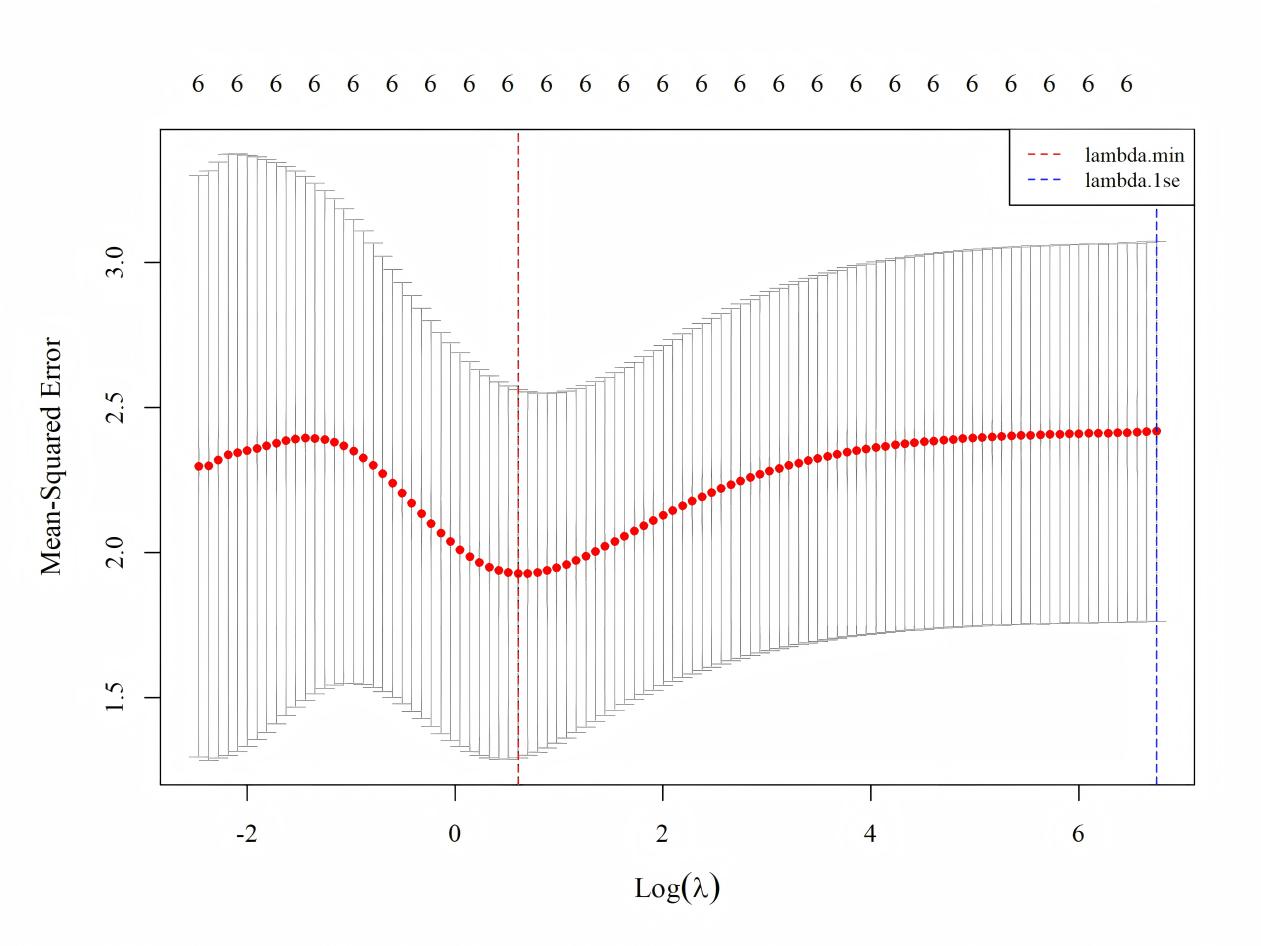


**Figure S2** Mean Square Error (MSE) of Ridge Regression Cross Validation with Regularization Parameter λ (lambda).


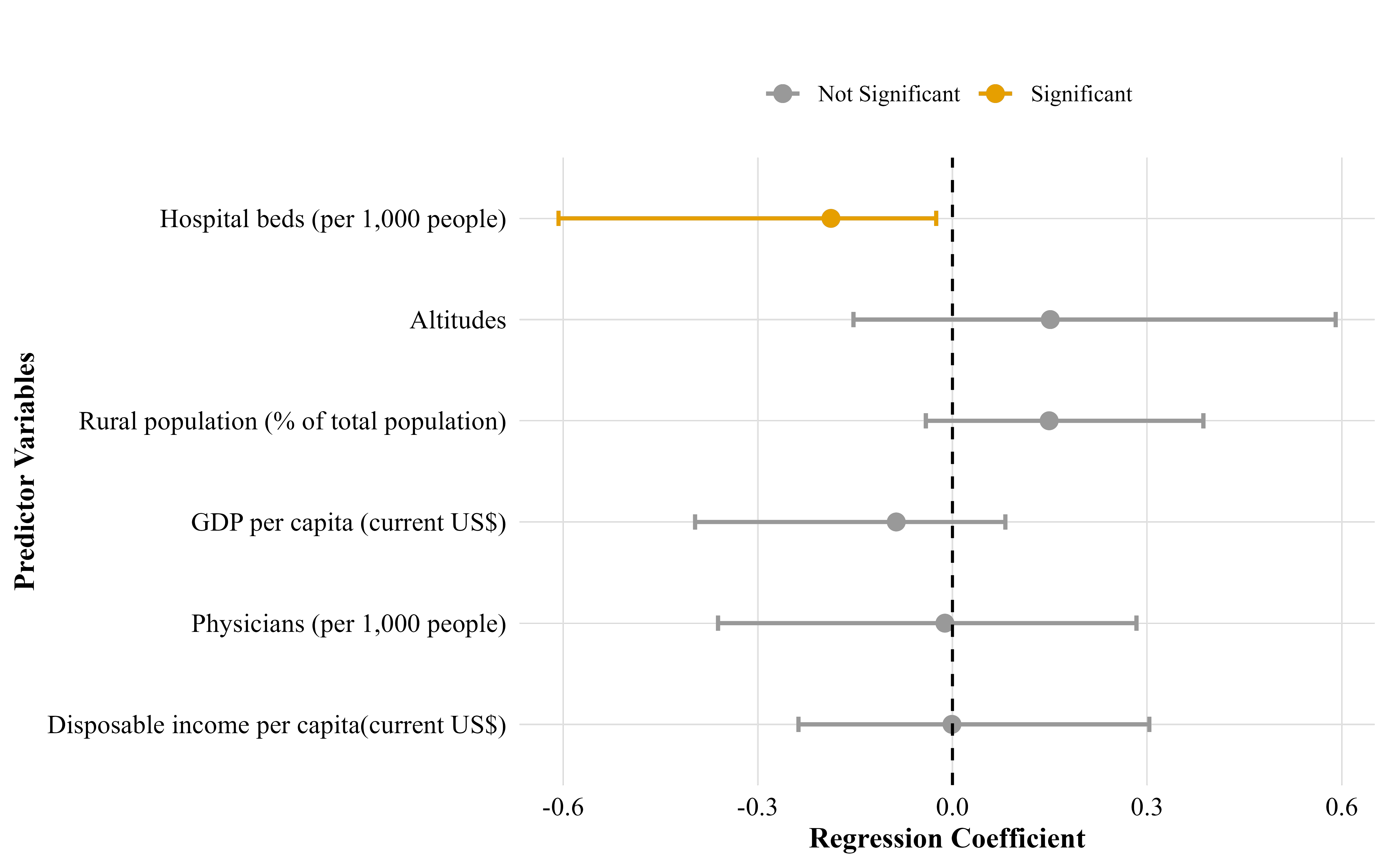


**Figure S3** Confidence intervals for ridge regression coefficients

**Supplementary Tables**

**Table S1** Prevalence of CHD, socio-economic health service capacity in different counties and cities.

|  | Prevalence  (per 1,000 people) | GDP per capita  (current US$) | per capita disposable income(current US$) | [Rural population (% of total population)](https://data.worldbank.org/indicator/SP.RUR.TOTL.ZS) | Hospital beds  (per 1,000 people) | Physicians (per 1,000 people) | Altitude |
| --- | --- | --- | --- | --- | --- | --- | --- |
| NEPAL | 5.99 | 1377.63 | 1005.68 | 78.00 | 0.39 | 0.87 | 2887 |
| YUNNAN |  |  |  |  |  |  |  |
| JIANGCHEN | 2.91 | 7986.77 | 4974.79 | 54.60 | 6.19 | 2.64 | 1197 |
| JINGGU | 2.54 | 7058.31 | 5292.09 | 66.75 | 6.59 | 1.28 | 1415 |
| ZHENGYUAN | 5.11 | 7264.22 | 4957.80 | 59.52 | 5.80 | 3.12 | 1598 |
| JINGDONG | 3.31 | 5613.90 | 4938.62 | 69.40 | 6.79 | 1.85 | 1788 |
| MOJIANG | 3.92 | 4750.11 | 4896.02 | 65.82 | 7.48 | 2.73 | 1498 |
| XINJIANG |  |  |  |  |  |  |  |
| AKESU | 3.48 | 7669.79 | 5220.80 | 40.10 | 3.56 | 1.66 | 1188 |
| WENSU | 6.62 | 6409.58 | 5258.16 | 68.76 | 3.33 | 1.26 | 2391 |
| AWATI | 5.54 | 4850.91 | 5194.94 | 79.71 | 5.76 | 1.36 | 1069 |

**Table S2** Results of multiple linear regression of the effect of socioeconomic and health service capacity on the prevalence of CHD.

|  | estimate | std.error | statistic | *p* | Tolerance | VIF | R | R2 |
| --- | --- | --- | --- | --- | --- | --- | --- | --- |
| Intercept | -4.24 | 0.746 | -5.68 | 0.0296 |  |  | 0.997 | 0.990 |
| GDP per capita (current US$) | -0.000361 | 0.000071 | -5.04 | 0.0372 | 0.1240895 | 8.058702 |  |  |
| per capita disposable income(current US$) | 0.00148 | 0.000105 | 14.0 | 0.00505 | 0.1292299 | 7.738147 |  |  |
| Rural population (% of total population) | 0.106 | 0.00884 | 12.0 | 0.00692 | 0.2339225 | 4.274920 |  |  |
| Hospital beds (per 1,000 people) | -1.18 | 0.0578 | -20.4 | 0.00240 | 0.1591894 | 6.281824 |  |  |
| Physicians (per 1,000 people) | 1.63 | 0.109 | 14.9 | 0.00446 | 0.3693383 | 2.707545 |  |  |
| Altitude | 0.0000094 | 0.000158 | 0.0594 | 0.958 | 0.2935173 | 3.406954 |  |  |

**Table S3** Ridge regression coefficients and standardized regression coefficients for the effect of socioeconomic and health service capacity on the prevalence of CHD.

|  | regression coefficient | standardized regression coefficient |
| --- | --- | --- |
| Hospital beds (per 1,000 people) | -0.27454708 | -0.1482842 |
| Altitude | 0.22099691 | -0.1277574 |
| Rural population (% of total population) | 0.21853043 | -0. 1198771 |
| GDP per capita (current US$) | -0.12698036 | -0.08001346 |
| Physicians (per 1,000 people) | -0.01700760 | -0.02344682 |
| per capita disposable income(current US$) | -0.00110518 | -0.02041086 |

The following are the types of congenital heart disease included in this study. The ICD-10-CM codes for the diseases that need to be noted below include only those congenital heart disease types identified by this study screening, not all types of congenital heart disease:

**ICD-10-CM Code Ranges for CHD**

CHD codes fall under Q00-Q99 (Congenital Malformations).

Q20-Q28: Congenital malformations of the circulatory system

**Table S4** This study screened for the types of children with congenital heart disease and ICD-10-CM Code

| Diagnosis | ICD-10-CM Code |
| --- | --- |
| Patent foramen ovale(PFO) | Q21.101 |
| Atrial septal defect(ASD) | Q21.100 |
| Ventricular septal defect(VSD) | Q21.000 |
| Aortic regurgitation (AR)‌ | Q23.100 |
| Patent ductus arteriosus(PDA) | Q25.000 |
| Pulmonary stenosis(PS) | Q22.100 |
| Mitral valve prolapse(MVP) | Q23.600 |
| Mitral valve stenosis(MS) | Q23.200 |
| Tricuspid stenosis(TS) | Q22.400 |
| Tetralogy of Fallot(TOF) | Q21.400 |
| Dextrocardia | Q24.000 |
| Pulmonary atresia (PA) | Q25.500 |
